# Pigmented Corneal Ulcer

**DOI:** 10.18502/jovr.v14i4.5461

**Published:** 2019-10-24

**Authors:** Sonam Yangzes, Parul Chawla Gupta, Vivek Jha, Jagat Ram

**Affiliations:** ^1^Department of Ophthalmology, Post Graduate Institute of Medical Education and Research, Chandigarh, India

**Keywords:** Corneal Ulcer, Keratitis, Pigmented

## Abstract

**Purpose:**

To report the clinical characteristics, laboratory findings, and treatment of a rare case of keratitis caused by pigmented fungi *Bipolaris hawaiiensis.*

**Case Report:**

A 55-year-old man presented with a history of trauma with vegetative matter in his left eye. Slit lamp biomicroscopic examination revealed the presence of a brownish-black pigmented plaque with surrounding infiltrates. Corneal scrapings revealed multiple septate hyphae. Culture revealed growth of the *Bipolaris* species. The patient was treated with topical natamycin 5%, topical voriconazole 1%, and oral itraconazole followed by intracameral amphotericin B (5 μg/mL). The patient responded well to the treatment.

**Conclusion:**

Brown pigmented infiltrates are an important clinical feature of dematiaceous fungi. *B. hawaiiensis* is a rare cause of corneal phaeohyphomycosis. Our patient responded well to intracameral amphotericin B, which obviated the need for penetrating keratoplasty.

##  INTRODUCTION

Fungal keratitis is one of the most common causes of keratitis in tropical countries.^[1,2]^ Dematiaceous fungi are the third most common cause of keratomycosis, with *Curvularia* and *Bipolaris* being the most common infecting species.^[3,4]^

*Bipolaris hawaiiensis* is a darkly pigmented fungus, widely distributed in nature, that causes cutaneous and soft tissue diseases known as phaeohyphomycosis;^[5]^ it is an extremely uncommon cause of keratitis. Oral itraconazole and topical natamycin have been used for this condition, while a few case reports have described additional use of topical amphotericin B in refractory cases. We report a case of keratomycosis, caused by *B. hawaiiensis,* that was treated with intracameral amphotericin B and showed a good response.

**Figure 1:**
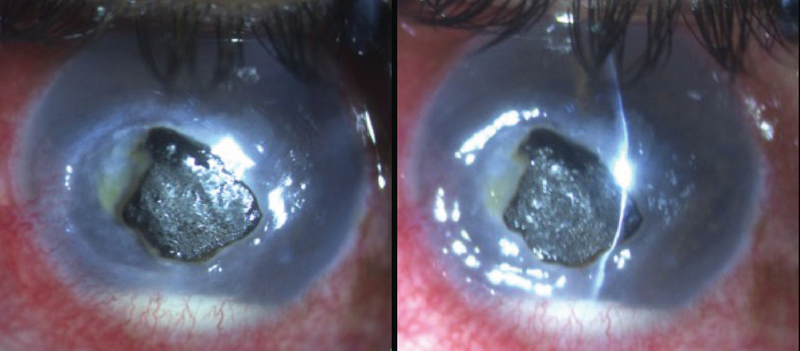
(a) A corneal ulcer measuring 6.5 × 5.5 mm, with a central pigmented plaque 4 × 4 mm with hypopyon. (b) Slit view showing area of corneal thinning.

**Figure 2:**
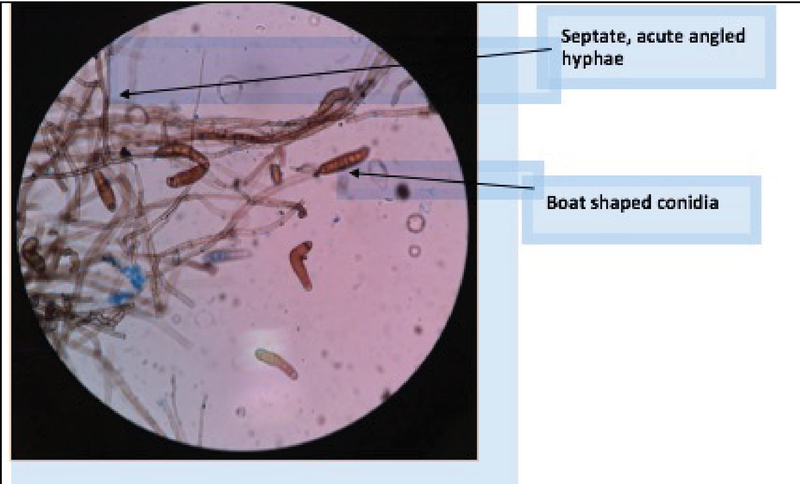
Lactophenol cotton blue staining showing acute angled septate hyphae with boat-shaped conidia.

**Figure 3:**
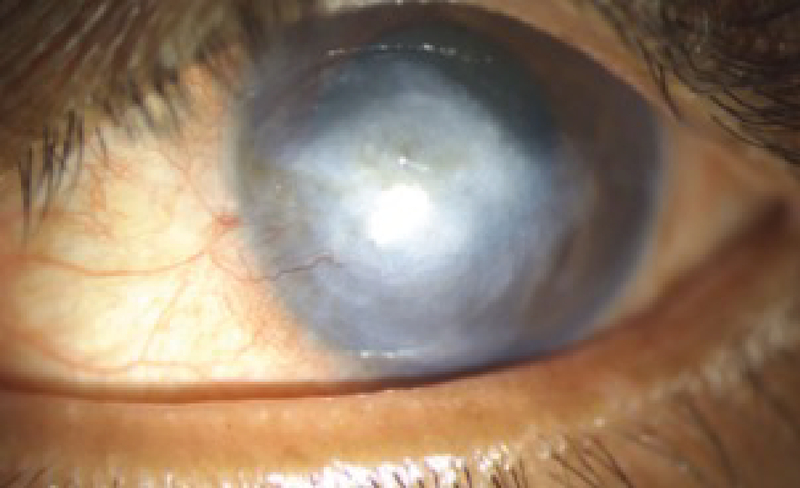
Healed ulcer with central scarring and peripheral vascularization.

##  CASE REPORT

A 55-year-old male farmer with a history of trauma to the left eye with vegetative matter presented at the ophthalmology clinic with complaints of diminution of vision, redness, photophobia, and black discoloration of his left eye. The visual acuity in the right eye was 6/6, while vision in the affected eye was restricted to the perception of light.

Slit lamp biomicroscopy revealed conjunctival congestion, a central pigmented corneal ulcer, and hypopyon. There were no other predisposing factors such as diabetes or other immunocompromised states. The central ulcer measured 6.5 x 5.5 mm, and showed a pigmented, elevated brownish-black plaque measuring 4 x 4 mm, with surrounding infiltrates. The ulcer appeared dry, with the presence of immobile hypopyon [Figures 1(a) and (b)]. The right eye presented with a clear cornea, early cataract, and a normal fundus. The plaque was scraped off and used to inoculate culture media. Due to the typical appearance of the ulcer (dry, pigmented plaque), we initiated hourly topical antifungal natamycin 5% treatment along with two-hourly topical moxifloxacin 0.5%, and cycloplegic and lubricating eyedrops. The culture revealed growth of the *Bipolaris* species. Lactophenol cotton blue staining showed acute-angled septate hyphae with boat-shaped conidia [Figure 2]. The hypopyon remained refractory to treatment after which amphotericin B (5 μg/mL) was injected intracamerally. The infiltrates became organized and the hypopyon disappeared. The lesion healed completely with central scarring and vascularization [Figure 3]. The final visual acuity was finger counting close to the face with accurate projection of light.

##  DISCUSSION

The *Bipolaris* species is classified as a dematiaceous or darkly pigmented fungus that causes phaeohyphomycosis, rarely infecting humans. The most frequently reported species are *B. spicifera, B. australiensis*, and *B. hawaiiensis*.^[6]^ Anadi et al have reported corneal ulcers due to *Bipolaris spp*. infection in a leprosy patient,^[7]^ while Bashir et al have reported keratomycosis with endophthalmitis in an immunocompetent individual.^[8]^ *B. hawaiiensis* is widely distributed in plants or soil and is commonly found in tropical regions. Dematiaceous fungal keratitis presents typically as recalcitrant-pigmented plaques that prevent the penetration of drugs, making superficial keratectomy essential in many cases.^[9]^ Culture shows dark septate hyphae with numerous conidia of the *Bipolaris* species. Although, the use of topical natamycin has been reported to be successful in treating most cases of dematiaceous fungal keratitis,^[3]^ our case responded well to intracameral amphotericin B injection, which obviated the need for long-term use of oral antifungal drugs as well as therapeutic penetrating keratoplasty.

##  Financial Support and Sponsorship

Nil.

##  Conflicts of Interest

There are no conflicts of interest.

## References

[1] Chowdhary A., Singh K. (2005). Spectrum of fungal keratitis in North India. *Cornea*.

[2] Tanure M. A., Cohen E. J., Sudesh S., Rapuano C. J., Laibson P. R. (2000). Spectrum of Fungal Keratitis at Wills Eye Hospital, Philadelphia, Pennsylvania. *Cornea*.

[3] Garg P., Gopinathan U., Choudhary K., Rao G. N. (2000). Keratomycosis: Clinical and microbiologic experience with dematiaceous fungi. *Ophthalmology*.

[4] Gopinathan U., Garg P., Fernandes M., Sharma S., Athmanathan S., Rao G. N. (2002). The epidemiological features and laboratory results of fungal keratitis: a 10-year review at a referral eye care center in South India. *Cornea*.

[5] Ajello L., Georg L. K., Steigbigel R. T., Wang C. J. (1974). A case of phaeohyphomycosis caused by a new species of Phialophora. *Mycologia*.

[6] da Cunha K. C., Sutton D. A., Fothergill A. W., Cano J., Gene J., Madrid H., De Hoog S., Crous P. W., Guarro J. (2012). Diversity of Bipolaris Species in Clinical Samples in the United States and Their Antifungal Susceptibility Profiles. *Journal of Clinical Microbiology*.

[7] Anandi V., Suryawanshi N., Koshi G., Padhye A., Ajello L. (1988). Corneal ulcer caused by
*Bipolaris hawaiiensis*. *Medical Mycology*.

[8] Bashir G., Hussain W., Rizvi A. (2009). Bipolaris hawaiiensis Keratomycosis and Endophthalmitis. *Mycopathologia*.

[9] Garg P., Vemuganti G. K., Chatarjee S., Gopinathan U., Rao G. N. (2004). Pigmented Plaque Presentation of Dematiaceous Fungal Keratitis. *Cornea*.

